# Targeting a Silent Disease: Vascular Calcification in Chronic Kidney Disease

**DOI:** 10.3390/ijms232416114

**Published:** 2022-12-17

**Authors:** Catarina Marreiros, Carla Viegas, Dina Simes

**Affiliations:** 1 Centre of Marine Sciences (CCMAR), University of Algarve, Campus de Gambelas, 8005-139 Faro, Portugal; 2 GenoGla Diagnostics, Centre of Marine Sciences (CCMAR), University of Algarve, Campus de Gambelas, 8005-139 Faro, Portugal

**Keywords:** chronic kidney disease, vascular calcification, biomarkers

## Abstract

Chronic kidney disease (CKD) patients have a higher risk of developing early cardiovascular disease (CVD). Although vascular calcification (VC) is one of the strongest predictors of CVD risk, its diagnosis among the CKD population remains a serious clinical challenge. This is mainly due to the complexity of VC, which results from various interconnected pathological mechanisms occurring at early stages and at multiples sites, affecting the medial and intimal layers of the vascular tree. Here, we review the most used and recently developed imaging techniques, here referred to as imaging biomarkers, for VC detection and monitoring, while discussing their strengths and limitations considering the specificities of VC in a CKD context. Although imaging biomarkers have a crucial role in the diagnosis of VC, with important insights into CVD risk, circulating biomarkers represent an added value by reflecting the molecular dynamics and mechanisms involved in VC pathophysiological pathways, opening new avenues into the early detection and targeted interventions. We propose that a combined strategy using imaging and circulating biomarkers with a role in multiple VC molecular mechanisms, such as Fetuin-A, Matrix Gla protein, Gla-rich protein and calciprotein particles, should represent high prognostic value for management of CVD risk in the CKD population.

## 1. Introduction

Cardiovascular disease (CVD) is the leading cause of morbidity and mortality globally, with chronic kidney disease (CKD) a significant contributor to this number. It is estimated that over 850 million people worldwide have kidney disease [1]. CKD is a prognostic comorbidity of COVID-19, and currently its prevalence is twenty times higher than the prevalence of cancer [2] and the number of individuals affected by the acquired immune deficiency syndrome (AIDS) [3] worldwide. These numbers point towards a truly concerning scenario, indicating CKD as an unquestionable global public health priority.

Chronic kidney disease is intrinsically associated to cardiovascular events, where accurate prediction of cardiovascular risk is of major relevance for both disease prevention and progression [4].

Cardiovascular risk consists of a sum of factors, in particular, habits, behaviors, circumstances or conditions, reported to increase a person’s risk of developing cardiovascular disease. These traditionally rely on scoring tools that include demographic and clinical characteristics such as the Framingham Score and the European Heart Score guidelines [5]. However, identifying CVD risk and predicting future cardiovascular events for a specific CKD patient based on these guidelines may not be adequate. Even though two new score algorithms (SCORE2 and SCORE2-OP) have been recently published [6,7], these scoring tools still fail in recognizing the cardiovascular risk of a proportion of individuals, particularly in early developmental stages of CKD where CVD remains silent [8,9].

The need for a more suitable risk stratification for the CKD population has caused researchers to focus their attention on the importance of non-traditional factors, such as cardiovascular calcification, which can occur at multiple locations in the vascular tree, such as the myocardium, heart valves and vasculature. In particular, vascular calcification (VC) is one of the strongest predictors of cardiovascular risk and is a driver of disease progression among these patients [10], posing an increased risk for cardiovascular events [11].

However, detecting VC can be quite a clinical challenge, because it is a silent condition that can develop over several years without expressing any signs or symptoms [12], explaining why until recently it has been considered an exclusive age-related process and not addressed as a serious medical condition [13]. Over the last years, powerful imaging techniques (imaging biomarkers) have served as a support to refine this non-traditional cardiovascular risk through adequate scoring tools obtained from direct visualization of subclinical VC, as suggested by the latest European (EAPC) guidelines on cardiovascular disease prevention [14]. However, the complexity and nature of VC associated with CKD, particularly the multiple and co-existent locations in the vascular tree, and the relevance of detecting initial phases of calcification are major challenges for the exclusive use of imaging biomarkers as diagnostic and prognostic VC markers.

To complement clinical information given by imaging biomarkers, the use of circulating biomarkers, especially in a CKD context, is shaping the way how VC is clinically detected, allowing a more accurate evaluation of CVD risk [15]. Currently, some circulating biomarkers, mostly linked to inflammatory pathways (recommended by EAPC), are integrated in the clinical routine for CVD risk assessment, such as apolipoprotein-B-containing lipoproteins or C-protein reactive (CPR) [14]. Additionally, many agents have been suggested as potential biomarkers for VC, mainly due to their involvement and relevance in VC molecular mechanisms, with a role in its initiation and progression, providing information on the pathophysiologic mechanisms involved in VC, eventually allowing early detection and more accurate management.

In this review we discuss current and recently developed imaging techniques to detect and quantify VC, integrated with the diagnostic and prognostic value of circulating biomarkers that reflect not only VC status but also insights into novel therapeutic targets in CKD patients. We propose that individuals with enhanced risk of cardiovascular events in the CKD population may benefit from the use of a multi-modality approach that includes both circulating and imaging biomarkers to reflect the different pathophysiological pathways. A combined strategy comprising both approaches may help achieve better diagnostics in determining a true CVD risk for renal patients, adding value to patient care.

## 2. Vascular Calcification and Cardiovascular Clinical Outcomes

Vascular calcification (VC), defined as the mineral deposition of calcium phosphate crystals mainly in the form of hydroxyapatite in the vasculature, is nowadays recognized as an independent predictor of vascular morbidity and mortality in CKD [16], manifesting through coronary artery disease, heart failure, arrhythmias, angina or sudden cardiac death [17,18]. It can be observed in arteries of all sizes and can take place in the intima and/or the media layer of the vessels [19].

**Figure 1:**
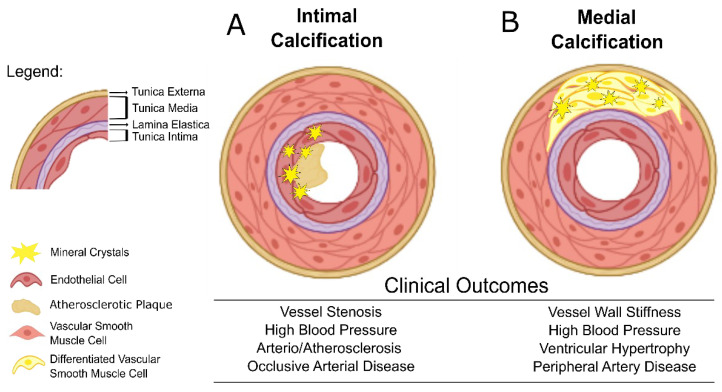
Intimal and Medial Calcification. (**A**) Intimal calcification is confined to the lumen or endothelium of the vessel mainly associated with atherosclerosis, contributing to vessel stenosis, lumen narrowing and increased blood pressure. (**B**) Medial calcification affects the medial layer of the vessel wall mainly composed of vascular smooth muscle cells, leading to increased vessel wall stiffness with compromised blood pumping and compliance, reflecting in high blood pressure, peripheral artery disease or even ventricular hypertrophy.

Briefly, intimal calcification is associated with atherosclerotic lesions, occurring predominantly in large arteries and characterized by a vicious proinflammatory cycle. Advanced atherosclerotic plaques are characterized by the presence of VSMCs, macrophages, T lymphocytes, dendritic and mast cells, together with a dense accumulation of extracellular lipids, collagen forming a necrotic core with a fibromuscular layer facing the lumen and a contribution to lumen narrowing [12]. Deposition on the surface of the intima layer of small calcium crystals often denominated as microcalcifications, is an early event in the atherosclerotic plaque formation, and is associated with a thin fibrous cap leading to plaque instability and vulnerability. Rupture of vulnerable plaques results in red cell-rich necrotic core material release, that once in circulation may cause life-threatening coronary thrombosis [20]. Most of the acute CVD events, such as myocardial infarction and stroke, are described to be associated with microcalcifications present in thin fibrous caps of atherosclerotic plaques. Macrocalcifications in more advanced atherosclerotic plaques are usually associated with thick plaque caps and have been suggested as providing plaque mechanical stability and protection against plaque rupture. However, since macrocalcifications are bigger in size, there is an increased risk for thrombus formation, resulting in a local occlusive disease [21,22] (Figure 1A). Interestingly, a recent study evaluating the impact of CKD (eGFR < 60 mL/min/1.73 m^2^) on coronary atherosclerotic plaque composition and morphology concluded that this population has more extensive and severe atherosclerosis remaining in their coronary tree comparing with non-CKD patients [23]. Another study showed that the plaque composition of 78 CKD patients changed from necrotic core-rich to extensively calcium-rich plaques as renal function decreased [24]. Such data reveal that intimal calcification in a CKD context suffers tremendous morphologic changes with its mineral composition varying according to CKD stages.

Medial calcification, also known as Monckeberg’s sclerosis is described by diffuse calcium phosphate mineral deposits along the *lamina elastica* in the medial layer of the arterial wall, resulting in a typical “ring-track” often seen in plain X-ray imaging. Although occurring in large arteries, it is frequently found in small to medium sized vessels. This type of vascular calcification can also cause substantial luminal narrowing with reduced vessel elasticity and compliance [18] (Figure 1B), increased pulse pressure and left ventricular hypertrophy, eventually resulting in heart failure. Medial calcification is predominant in CKD patients when compared to non-CKD subjects [25]. Despite divergent data from London and colleagues [26], reporting longer survival for ESRD patients with medial calcification compared to patients with intimal calcification, medial calcification diagnosis has been shown to have a higher prognostic value for identifying CKD patients at high cardiovascular risk [15,27].

However, medial and intimal calcification often coexist in CKD patients, enhancing each other with the potential overlap of pathological processes and clinical outcomes [12,15]. Such features pose additional challenges to accurately localize the mineral deposits, as will be discussed below.

## 3. Imaging Cardiovascular Calcification in CKD Patients

Over the past decades, imaging methods, henceforward referred to as imaging biomarkers, have played a crucial role in the diagnosis of VC, with important insights into cardiovascular risk. One of the reasons that may explain the struggle for accurate VC diagnosis in CKD patients is that the calcification characteristics change alongside renal deterioration and CKD progression [23,24]. Here, we summarize current and recently developed imaging techniques for vascular calcification in a CKD context, their applications in clinical research (Table 1) and discuss their suitability according to VC development. We approach radiology, ultrasonography and computed tomography (CT) scan techniques, and their respective VC scores frequently used in the clinical routine for CKD patients. At the same time, we discuss current evidence-based indications relevant to integration of novel imaging biomarkers in the CVD risk assessment in CKD.

### 3.1. Radiology Techniques

Conventional X-rays can identify extra-skeletal calcifications, particularly in the aorta and peripheral arteries. Radiographs have the advantage of being economical, with less exposure to radiation compared to CT scans. They seem to provide valid prognostic information scores, as well as simple and readily available clinical data, representing a good method to screen for the presence of VC in CKD patients. The KDIGO (kidney disease: improving global outcomes) guidelines from 2017 [45] recommend radiologic methods as evaluation tools for VC diagnosis, particularly the lateral lumbar spine X-ray for VC assessment in stages 3–5 D. In concordance, several observational studies involving CKD populations have confirmed that using plain X-rays in different body locations is an effective alternative for VC detection and CVD risk evaluation, either in early stages of the disease [46] or in a hemodialysis population [47].

Similar to ultrasonography and computed tomography, plain X-rays also have specific scoring tools that can give physicians a semi-quantitative analysis of calcium deposits. In 2004, Teresa Adragão et al. developed a simple score to assess subclinical vascular disease in a prospective observational study involving 123 hemodialysis patients [28]. The Adragão score, obtained by a plain X-ray of the hands and pelvis (iliac, femoral, radial and digital arteries), is not only a tool for the assessment of cardiovascular risk, but also an indicator associated with arterial stiffness and mortality in dialysis patients [48]. The Adragão score is suitable for detection of heavy deposits of mineral crystals in the media layer of the vessels; the most common type of calcification affecting small vessels and typically found among CKD patients. These deposits localized in peripheric arteries are recognized to have a high CVD risk prognostic value for CKD patients [27]. Another X-ray-based score was established by Kauppila et al. [30] from a retrospective study involving 617 lateral lumbar radiographs performed on patients from the Framingham heart study. The Kauppila score, representing the abdominal aorta calcification score (AACS) [49], assesses vascular calcifications via lateral abdominal X-rays and was proven to be a simple, low cost assessment of subclinical vascular disease (Table 1). More recently, it was correlated with cardiovascular event prediction in a study involving 187 peritoneal dialysis patients, and was showed to improve risk stratification beyond the Framingham risk score in another study involving 184 hemodialysis patients [50]. Observational studies comparing the performance of both the Adragão and Kauppila scores with the CT-scan based score (Agatston) support the notion that X-ray-based calcification scores are valid alternatives to CT scans, with incremental prognostic values for CV events beyond traditional risk factors. One of the studies, involving 143 patients at various stages of CKD, showed a significant and linear relationship between the Agatston and Kaupilla scores [51]. In addition, a cross-sectional study performed in 50 hemodialysis patients showed a correlation between the Adragao and Agatston scores to clinically detect vascular calcification for this population [52].

Nevertheless, X-ray imaging techniques still include drawbacks and limitations, such as they allow a clear identification of VC lesions only when the vessel is considerably calcified [53]; difficulties in distinguishing between medial and intimal calcification if they coexist in the vessel; the semi-quantitative nature of the calcification reading due to the inability to precisely quantify calcium deposits; and the inability to reliably detect subtle temporal changes in VC. These pose major challenges for early VC diagnosis and disease progression monitoring, limiting its use for CVD risk detection in early stages of CKD—stages 1 and 2, where the disease is in most cases silent—highlighting a clear need for complementary clinical exams.

### 3.2. Ultrasonography Techniques

In conventional ultrasonography methods, their resolution is dependent on the depth of area, which makes them appropriate for detection of calcification deposits in superficial arteries (carotid, femoral and peripheral arteries). Ultrasonography has the benefit of no radiation exposure and is a relatively economical choice, widely available at most health care facilities, and has the potential to be implemented as a routine method for VC detection. Since it specifically analyzes the wall thickness and lumen size, it can detect stenosis of the arteries and mineral deposition sites either in vessels or cardiac valves [50,51]. Some studies have suggested the more efficient ability of ultrasonography to detect medial calcification compared to the X-ray approach [53], by it clearly being able to distinguish between the different layers of the arterial wall, with the advantage of also detecting calcium deposits in aortic and mitral valves. Since medial calcification is prevalent in CKD patients, ultrasonography, and its associated VC scores, appears to be a good method to assess VC in the CKD population [33,53,54,55].

Several calcification scores based on ultrasound detection of calcium deposits have showed the potential of this technique to detect VC in early or even asymptomatic CVD patients at risk (Table 1). Although not yet deeply studied in the CKD population, some of these scores can be particularly relevant for CKD at earlier stages where CVD diagnostic is particularly challenging [56,57]. The transthoracic echocardiography-based score (Echo score) [34] showed strong correlation with coronary artery disease (CAD) and association with the CT-scan based score (Agatston) in non CKD patients with low/intermediate CVD risk [58]. The color Doppler ultrasound score (CALCs) [59] was found to be highly correlated with markers of subclinical atherosclerosis, such as the intima–media thickness and arterial stiffness in asymptomatic patients at low–intermediate CVD risk, predicting CVD events beyond the traditional risk factors [35]. The duplex ultrasound (DUS)-based score, named DULLAC, assessed the lower limbs artery calcification of six patients selected randomly from the Vascular Studies Unit at Cambridge University Hospitals and added an input to risk stratification in non-CKD patients with peripheral arterial disease [32] by correlating with CT-based arterial calcium measurements.

More recently, intravascular modality based on wave-sound, called intravascular ultrasound (IVUS), has revolutionized ultrasonography methods by detailing plaque and vessel wall in real-time throughout the coronary artery tree. IVUS has been largely used due to its sensitivity and specificity for detecting calcium within a plaque, enabling calcium deposit localization and distribution, as well as a more precise quantification of calcification than the previous described ultrasound techniques [36]. A study comprising 440 patients with stable angina aimed to compare IVUS, optical coherence tomography (OCT) and coronary angiography in their ability to target lesion calcification in patients undergoing percutaneous coronary intervention. The results showed that calcium deposits were detected by IVUS in 83% of patients, OCT in 77% of patients and angiography in 40% of patients, revealing a higher accuracy of the IVUS method in detecting calcifications. This ultrasound modality score [36], designated as the IVUS calcium score, was proposed as a robust and accurate tool to assess both the presence and amount of coronary calcification.

Although promising for predicting CVD risk, these ultrasonography scores still lack specific evidence for CKD patients, requiring further studies to validate its use for routine assessment among this population. Additionally, due to its invasive nature, the IVUS method should be used with precaution to avoid disturbance of existing unstable plaques.

Even though ultrasonography can add substantial input to VC diagnosis, it still reflects some limitations regarding evaluation of calcium mineral deposition. Current ultrasonography methods, except for IVUS, lack on providing a quantification of mineral deposits in vessels and valves [60]. Furthermore, these ultrasonography techniques still fail on providing detection of microcalcifications in atheromatous plaques, and are fit only for diagnosis of macrocalcifications.

### 3.3. Molecular Imaging Techniques

Computed tomography (CT) scanning is the gold standard method for evaluation of VC, allowing an objective quantification of calcium deposits. CT scanning is a non-invasive exam using X-rays for imaging of the heart and its structures.

Computed tomography is very useful for cardiovascular risk stratification by providing a reliable quantification of macroscopic calcium deposits expressed as a clinical coronary artery calcium score (CACS) [61] or aortic valve calcification score (AVCS) [62]. The importance of CACS evaluation is evidenced by the latest 2019 guidelines of the American College of Cardiology/American Heart Association, which included for the first time “Coronary Artery Calcium” as an assessment tool for cardiovascular risk in individuals with intermediate predicted risk [63]. CACSs to stratify the cardiovascular risk of their patients, and monitor disease progression: (1) the Agatston score [41], (2) volume of calcium score [64] and (3) calcium mass score [65]. Mostly due to its simplicity, the score proposed by the cardiologist Arthur Agatston in 1990 continues to be the reference CACS used for most population-based studies involving CVD risk stratification. These CAC-based scores can be used to detect calcification not only in carotids arteries, but also in larger arteries such as the abdominal [41,63] or thoracic aorta [38], and even in cardiac valves [66] (Table 1). 

In addition, large cohort studies [38,39,40] have demonstrated the ability of CACS to predict cardiac events in asymptomatic subjects, supporting an increment value over conventional risk factor assessment. In a CKD scenario, the extent of vascular and valvular calcification increases with renal deterioration throughout the disease stage progression. These CT scoring tools become important because the information provided by CAC score assessment can significantly alter clinical decisions, providing a closer disease monitoring with the possibility to intervene to delay VC progression [67,68,69]. In a large study of CKD patients as part of the Chronic Renal Insufficiency Cohort trial, the Agatston score was evaluated in 1541 CKD patients from stage two to four without established CVD [37]. In these patients, the CAC score was shown to improve risk prediction for cardiovascular disease, myocardial infarction and heart failure relative to established cardiovascular disease risk factors, highlighting the clinical input that the CAC score can add to medical care.

However, CT scans cannot accurately differentiate between the intimal and medial location of calcium deposits due to low resolution captured by the scan, challenging the detection of medial calcification in small arteries, which is characteristic in CKD. Moreover, the high equipment cost, the high-dose ionizing radiation exposure, the risk of nephropathy due to the contrast agent when coupled with angiography and the inability to perform in-office testing add unfavorable circumstances for the regular use of this method to assess VC, particularly in renal disease patients. Additionally, medial artery calcification does not always develop proportionally to obstructive coronary artery disease (CAD), limiting the prognostic value of CT scanning coupled with angiography [70]. Another major disadvantage of CT scans is that they only provide quantification of macrocalcifications. Although CT scans are considered good markers of overall CVD burdens, their inability to detect microcalcifications also argues their use in identifying vulnerable lesions prone to rupture, often characterized by the presence of microcalcifications in early phases of VC development.

Recently, other molecular imaging techniques have been explored as an alternative to CT scans, aiming to explore this flaw of lack of detection of microcalcifications. In CKD, a continuum of calcification typically occurs with evidence of both micro- and macro-calcification in the intima and media. However, X-rays, ultrasonography (except for IVUS) and CT-scans can only detect macrocalcifications. A solution for detecting microcalcification is reported in a recent technique called positron emission tomography (PET). This remarkable tool uses a radioactive tracer ((18) F-NaF) to detect small calcium deposits on the endothelium surface. A deeper description of the method can be found elsewhere. The use of PET fosters new approaches for diagnosis of microcalcification in the vascular tree, adding a clinical possibility to distinguish between areas of macro- and micro-calcification [71]. However, this technique is not yet commonly applied for clinical monitoring of cardiovascular disease in CKD patients, being mostly used for microcalcification diagnosis for several forms of cancer or for CVD in general [72].

Overall, due to the lack of sensitivity in distinguishing between calcification of the intima and media layers and limitations in detecting microcalcifications, CT scans might not always be the best method to assess VC in a CKD context. In light of these limitations, radiology and ultrasonography methods, under some circumstances, might be preferable choices over CT scans for CVD risk stratification in early CKD stages. However, they also have considerable restrictions for cardiovascular risk prediction in CKD patients. The establishment of a multimodality-based diagnostic tool, using not only imaging but also circulating markers for VC, could overcome the individual gaps of each imaging technique. The possibility to integrate novel VC circulating biomarkers in this multimodality-based diagnostic approach, could not only reduce the medical exam cost burden, but also introduce complementary information on VC pathophysiological pathways with implications for the disease management.

## 4. Circulating Biomarkers of Vascular Calcification in CKD

Circulating biomarkers, such as high sensitivity C-reactive protein, apolipoprotein-B-containing lipoproteins-like very low density lipoproteins (VLDL) or low density lipoprotein (LDL) and cardiac troponin, have already been shown to play a crucial role in the diagnosis, risk stratification and clinical management of patients with several cardiovascular disease conditions including heart failure (HF) [73] and acute coronary syndrome (ACS) [74]. Additionally, novel circulating biomarkers related to VC pathophysiological pathways have been associated with cardiovascular risk [75], representing encouraging options to provide important CVD prognostic information, by representing a more direct and unique reflection of the molecular dynamics involved in VC mechanisms.

In the particular case of CKD, VC is a multifactorial and highly regulated process, involving multiple, interconnected molecular mechanisms, such as dysregulation of endogenous calcification inhibitors, chronic inflammation and abnormal mineral metabolism [76]. An imbalance between calcification promotors and inhibitors leads to a series of events, such as osteogenic differentiation of VSMCs and the formation of calcium-phosphate crystals (CaP) or mineral nucleation sites, which can occur in circulation in the form of soluble calciprotein particles (CPPs) in mineralized competent extracellular vesicles (EVs) and can be deposited in blood vessels. These CaP promote pro-inflammatory reactions that in turn increase pro-calcific responses, in a cycle where increased calcification fuels inflammation and vice versa. Adding to this complex scenario, altered mineral metabolism, often associated with CKD, increased levels of circulating calcium (Ca) and phosphate (P) and subjacent hormonal control dysregulation further challenge the control of CaP formation and consequent VC outcomes. In this context, throughout the literature, several players and pathways [18,77] enhancing and inhibiting VC, which are deeply connected with the mineral metabolism, have been suggested as potential biomarkers and therapeutic targets for this pathologic process [78] (Table 2). In the past decade, fibroblast growth factor 23 (FGF23) and its membrane protein coreceptor, αKlotho, have emerged as novel disease biomarkers with potential relevance in cardiovascular risk assessment [79,80]. Findings from epidemiological studies, both in the general population and in CKD, are remarkably consistent and demonstrate an association of the FGF23-Klotho axis with important clinical events related to mortality [81], cardiovascular disease [82] and inflammation [83].

While the utility of these potential individual circulating VC biomarkers has been subject to validation using imaging biomarkers in clinical studies (Table 3), the overall results suggest that the combination of several circulating biomarkers reflecting different VC pathways could translate to a more reliable reading of VC status. In this line, the calcification inhibitors Fetuin-A, Matrix Gla Protein (MGP) [92] and Gla Rich Protein (GRP) [93] have been shown to have a role in multiple molecular mechanisms involved in VC. Furthermore, these three proteins have been proposed to function, at least in part, in a protein complex with powerful anti-mineralization capacity [94]. The occurrence of such protein complexes has been identified in the above-mentioned circulating calciprotein particles (CPPs). CPPs are formed from the combination of CaP and several circulating proteins including mineralization inhibitors such as Fetuin-A, GRP and MGP. CPPs have been reported to act as active precursors of microcalcifications [95] through its direct deposition in vascular tissues and capacity to induce VSMCs osteochondrogenic differentiation, and also to induce pro-inflammatory reactions. This pathogenicity has been shown to be directly related to CaP mineral maturation state, which in turn is highly dependent on the presence of Fetuin-A [96], GRP [93] and MGP [92]. In normal physiological conditions, CPPs are considered as a mineral guardian with a role in stabilization, transport and recycling of water-insoluble CaP mineral in blood and suggested to take part in a calcification inhibitory mechanism in the human cardiovascular system.

Due to their relevance and promising clinical use, in this review, we will particularly focus on the role and biomarker utility of Fetuin-A, MGP and GRP, as well as the use of CPP measurements to reflect serum calcification inhibitory capacity as potential added value circulating VC biomarkers in CKD.

### 4.1. Fetuin-A, Matrix Gla Protein and Gla Rich Protein in Vascular Calcification in CKD: Potential Biomarker Utility

Vascular calcification is a process that must be actively inhibited, relying on the constant presence of functional calcification inhibitors. The combination of pro-calcific stimuli with the decrease in inhibitory mechanisms, such as Fetuin-A and the vitamin K-dependent proteins MGP and GRP, contributes to mineralization of the extracellular matrix [93].

Preclinical data have previously shown the involvement of vitamin K in VC at the tissue and circulating level. This is accomplished through the carboxylation of MGP and GRP in a post translational reaction catalyzed by the enzyme γ-glutamyl carboxylase using vitamin K as cofactor, which activates these proteins involved in vascular health. The role of vitamin K-dependent proteins (VKDP) in cardiovascular health is emerging as a relevant topic that is currently under the attention of the scientific community [114].

Additionally, vitamin K deficiency is frequently found in CKD [115,116,117], and measurement of circulating non-carboxylated and non-phosphorylated inactive forms of MGP (dp-ucMGP) has been widely used in clinical studies to evaluate vitamin K status. Some clinical studies also show that vitamin K therapeutics lower the levels of circulating dp-ucMGP [108,109,110]. A cross-sectional study enrolling 83 CKD stage 3–5 patients showed that plasma dp-ucMGP levels were positively associated with vascular calcification and CKD severity [118]. Studies of CKD showed an association between increased levels of dp-ucMGP with CAC score and medial calcification [107], all-cause/CVD mortality [119] and arterial stiffness [120] and suggest it as a candidate marker of cardiovascular risk [121] among this population.

Although there are still some controversial data regarding the clinical utility of MGP as marker for VC [122], overall, the inactive dp-ucMGP has been clearly demonstrated as a marker for vitamin K status, VC and CVD risk, not only in specific disease populations (CKD, diabetes) [107,120,123], but also in healthy cohorts [124].

Gla-rich protein (GRP), also known as the upper zone of growth plate and cartilage matrix associated protein (UCMA), is a VKDP with a dual capacity to function as an inhibitor of pathological calcification and an anti-inflammatory agent in the articular and cardiovascular systems [125]. GRP is involved in VSMC osteochondrogenic differentiation, mineralization capability of EVs and in the growth and maturation of CPPs [126]. Recent clinical studies in CKD patients showed an association between GRP and CKD progression and cardiovascular calcification, in particular in vascular [46,99,110] and valvular calcification [111]. This last clinical study showed, for the first time, an interesting correlation between low levels of GRP and magnesium and the assessed calcification score in the CKD patients [111]. While many in vitro and in vivo studies have identified magnesium as a protective factor in vascular calcification, low magnesium serum levels have been associated with increased CVD risk in the general population and low survival in end-stage renal disease [127]. Although containing less data from human studies than MGP, these findings suggest that GRP may have the potential to be used as a cardiovascular calcification marker in patients with CKD.

Fetuin-A is a glycoprotein synthesized in the liver released in the bloodstream and is known to be up taken by VSMCs. It acts as a mineral gatherer, binding CaP mineral in CPPs and preventing crystal growth and maturation, playing a pivotal role in inhibiting soft tissue calcification and local mineralization of the vascular wall [128]. Fetuin-A was shown to be predominant in CPP1 and less represented in CPP2 particles and widely suggested to stabilize CPP1 and retard the progression towards CPP2 [129]. Although some studies measuring total Fetuin-A point to the opposite [113], supporting data in patients with CKD suggest that total levels of Fetuin-A are inversely associated with endothelial disfunction [130] and were linked to inflammation and all-cause mortality in a dialysis population [131]. In addition, a meta-analysis including 13 clinical studies comprising 5169 CKD patients showed that low levels of serum Fetuin-A (total Fetuin-A) were associated with an increased risk of all-cause mortality [132].

### 4.2. Calciprotein Particles (CPPs) as a Potential Biomarker of Vascular Calcification Progression in CKD

CPPs have been particularly associated to CKD due to the characteristic disturbed mineral homeostasis, which leads to the passive formation of CPPs that aggregate the excessive circulating calcium and phosphate ions [133]. The progressive kidney function deterioration in CKD continuously leads to enhanced formation of CPPs, exceeding the body excretory capacity and aggravating pro-inflammatory and pro-calcific pathways [134].

**Figure 2:**
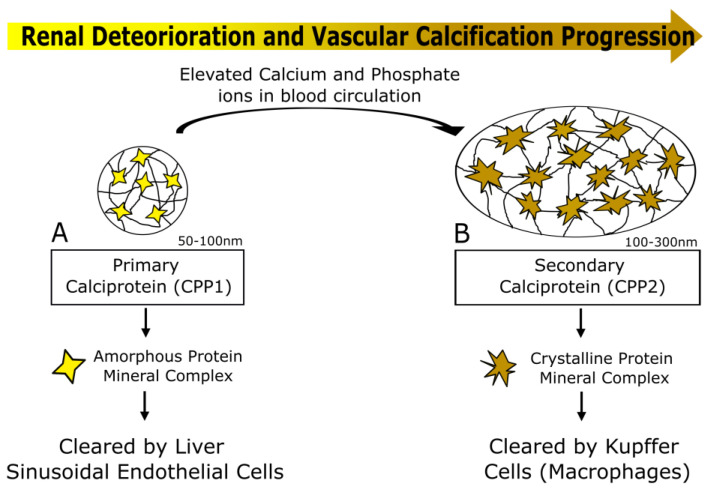
Calciprotein particle formation, maturation and clearance from circulation. (**A**) Primary calciprotein particles (CPP1) are circulating particles formed of a combination of CaP mineral and several mineral regulatory proteins including Fetuin-A, MGP and GRP, where the mineral is held in an amorphous shape. CPP1 are cleared by highly specialized liver sinusoidal endothelial cells. (**B**) Under conditions of hyperphosphatemia and hypercalcemia, as observed in mineral and bone disorder in CKD, CPP1 undergo an amorphous to crystalline transition into needle-shaped crystallike secondary CPPs (CPP2). CPP2 are described to be cleared by resident liver macrophages (Kupffer cells).

These calciprotein particles exist in two different phenotypes, primary and secondary CPP (CPP1 and CPP2, respectively), which differ in shape, function, crystal maturation and diameter. If not countered, the initially formed innocuous amorphous spherical complexes, CPP1, mature into the harmful needle-shaped crystalline secondary complexes, CPP2 (Figure 2). This transition marks a key trigger of both medial and intimal VC, because CPP2 containing hydroxyapatite can be internalized by VSMCs, promoting differentiation to an osteoblast-like phenotype (medial calcification) [135]. CPP2 can also induce endothelial damage by deposition in the ECM, representing the footings of microcalcification deposits in intimal VC [136]. This process is highly regulated by mineralization inhibitors such as Fetuin-A, MGP and GRP, reinforcing their importance as therapeutic targets for vascular calcification in renal disease patients. With the highlighting of the molecular mechanism of CPP formation, its composition and its importance in vascular calcification, several methodologies have been developed and proposed for measurement of serum CPP. Most of these methodologies are in vitro-based blood assays evaluating serum calcification capacity, either relaying on the indirect estimation of CPP-bound Fetuin-A, the CPP-Fetuin-A test [84], or on the capacity of blood to resist calcification, indirectly reflecting levels of calcification inhibitors, the T50 test [137]. These simple tests have been recently used in several clinical studies showing a highly promising clinical utility as markers for VC, CKD development and cardiovascular events in the CKD population (further detailed below). In addition, direct measurements of CPP-bound GRP and Fetuin-A in isolated serum CPPs showed that CPP from stage V CKD patients, with CPP2-like features and able to induce mineralization and inflammation in VSMCs, contained less GRP and Fetuin-A than CPP from healthy individuals [126]. This suggests that measurements of CPP-bound GRP, less explored than Fetuin-A, might add or complement VC clinical utility to total GRP as described in Section 4.1.

#### CPP-Fetuin-A and T50 Calcification and Tests

**Figure 3:**
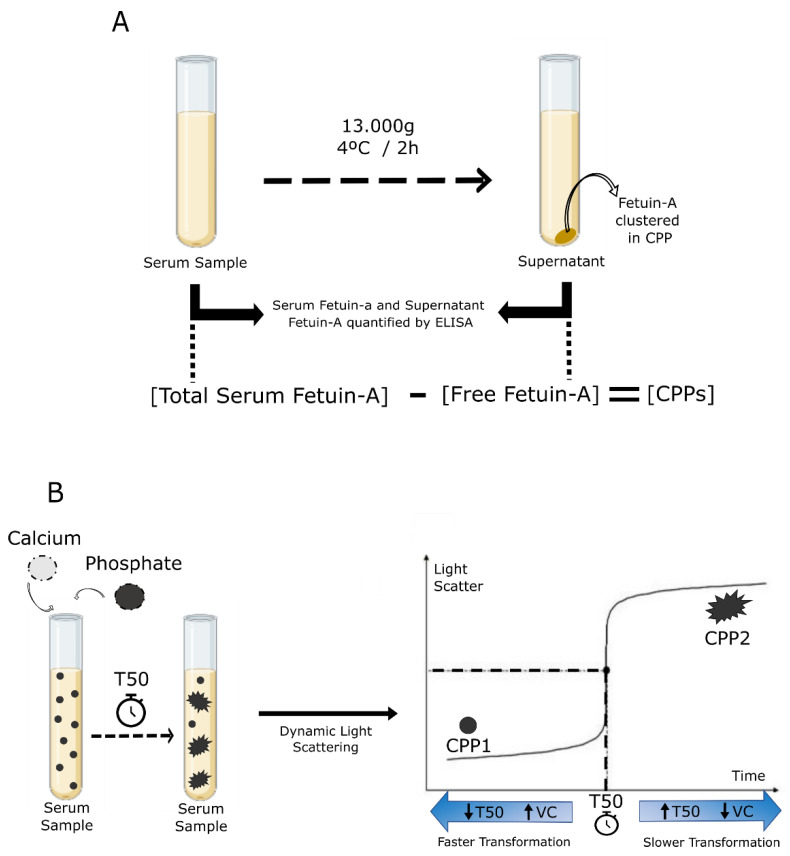
Calciprotein particle in vitro blood tests. (**A**) The CPP/Fetuin-A test measures the CPP concentration through a ratio between total Fetuin-A and non-sedimented Fetuin-A after centrifugation of serum samples. (**B**) The T50 test is a method that involves the mixing of patient serum with buffered solutions of calcium and phosphate and subsequent monitoring of changes in light scattering to determine the half time of transition (T50) from primary to secondary CPP in serum.

The CPP-Fetuin-A test is based on the capacity of Fetuin-A to interact with calcium and phosphate, generating CPPs. In brief, the amount of circulating CPP is indirectly determined by measuring Fetuin-A concentration by ELISA before and after centrifugation of serum samples. This reduction ratio, accounting for the difference between the total (free Fetuin-A + CPP-bound Fetuin-A) and the non-sedimented Fetuin-A (free Fetuin-A) is proposed to estimate CPP, based on the estimated CPP-bound Fetuin-A levels [84] (Figure 3A). The higher the CPP reduction ratio, the smaller the capacity of blood to resist calcification.

The T50 test determines the propensity of blood to resist calcification by measuring the calcification inhibitory potential in individual blood samples [137].

It represents the delay of the transformation of CPP1 to CPP2 generated ex vivo in serum samples, by adding very high concentrations of calcium and phosphate solutions. The test measures the duration of this CPP transformation by calculating the time point of the half-maximal generation of secondary CPPs (Figure 3B). Short T50 is consistent with low calcification resistance indicating accelerated transformation, while long T50 reflects a delayed transformation of CPP, associated with an intact calcification resistance system [137].

Clinical studies in several stages of renal disease draw attention to the relation between CPP and CKD population. A study comprising 200 patients in CKD stages 3 and 4 showed an association between increased CPP Fetuin-A levels and increased aortic stiffness [138]. An interventional study, enrolling 39 hemodialysis patients, used the T50 test to monitor the impact of the phosphate binder, sucroferric oxyhydroxide (SO), on serum phosphate levels. In high dose SO-treated patients, decreased phosphate serum levels were associated with increased T50, indicating an improved serum calcification propensity [139]. Data obtained using the T50 test have been found to correlate with relevant clinical events such as severity and progression of coronary calcification, aortic stiffness, cardiovascular and all-cause mortality in the CKD population [140,141]. A longitudinal cohort of 699 stable renal transplant recipients showed an association between fast CPP2 formation and increased risk of all-cause mortality and cardiovascular mortality [142].

Additionally, it is established that the serum of ESRD patients has a higher propensity to form CPP2 when compared with serum from healthy individuals [143]. Interestingly, studies with CKD and hemodialysis patients demonstrate that therapeutic interventions among these patients can be visualized with the T50 value [144,145]. Moreover, since T50 reflects the ability of a given serum to delay transition from CPP1 to CPP2, which is specific for each individual, it holds the potential for personalized therapy, adding value to patient care and current VC management. This test can be used as a practical tool by nephologists to complement patient clinical information, possibly alongside imaging VC scores, in a multimodality approach comprising circulating and imaging biomarkers.

## 5. Conclusions

Vascular calcification is nowadays an accepted non-traditional risk factor for cardiovascular risk assessment in the CKD population. While its diagnostic utility is unquestionable, the way it is diagnosed still faces many challenges in a CKD context, where VC is particularly complex, resulting from multiple interconnected pathological mechanisms. Ideally, VC should be detected at early CKD stages characterized by microcalcifications specifically localized at the media and/or intimal layers of the vessel walls, providing pathophysiological information allowing specific and targeted intervention strategies.

The use of imaging techniques to detect and monitor VC is imperative to assess CVD risk, but specific drawbacks are still associated with most imaging techniques, particularly for CKD patients. Although the choice of the most suitable technique may be dependent on several factors, it is established that CT scans are the gold standard to detect large calcium deposits. Nevertheless, it lacks the sensitivity to distinguish calcification of the intima and media layers, and has limitations in detecting microcalcifications, leaving space for X-rays and ultrasonography to add important information in a much faster and cheaper way about the localization of the mineral deposits. However, these two methods are only adequate for superficial arteries, and lack the ability to quantify the mineral deposits.

Despite the availability of different image-based techniques suitable for VC detection, CVD risk stratification and the applied VC scores are not yet standardized. This directly reflects in controversial data from different clinical studies in the literature. While there is still a need for guidelines to develop and use VC scores uniformly with reproducible CVD risk conclusions, VC circulating biomarkers can be useful tools to complement some of the existing clinical gaps, such as the status of the anti-calcification mechanisms of a given patient.

Circulating biomarkers, such as Fetuin-A, the vitamin K dependent proteins MGP and GRP and calciprotein particles, are known calcification inhibitory systems with roles in multiple molecular mechanisms known to be involved in the development and progression of vascular calcification. These features make them attractive targets to follow VC from early developmental stages, providing molecular information on pathophysiological mechanisms and opening the possibility of specific interventional strategies.

While there is still a broad research area to fully uncover aspects related to the roles and molecular interactions of these VC circulating biomarkers, mounting clinical evidence shows that they might be useful tools to improve early diagnosis, monitor progression or identify CVD risk in CKD patients. Physicians should stay conscious to the use of novel and promising VC circulating biomarkers as screening tools to assess CVD in CKD as a complement to patient clinical information in the near future. Accurate diagnosis and follow-up that influence decision-making can be improved with the combination of imaging and circulating biomarkers, arming physicians with useful clinical instruments.

## Figures and Tables

**Table 1:** Imaging Techniques and Scores for Cardiovascular Calcifications in the CVD and CKD Population.

Clinical Assessment	Anatomical Sites	Population	VC Scores	Refs.
**X-Ray** **Techniques**				
**Plain X-ray**	Hands, pelvis, femur	Hemodialysis patients	Adragão Score	[28,29]
**Lateral Dual-energy X-ray Absorptiometry**	Abdominal aorta artery	Dialysis patients	Kauppila Score	[30,31]
**Ultrasonography** **Techniques**				
**Duplex Ultrasound**	Common femoral artery, proximal and distal superficial femoral artery and popliteal artery	Peripheral arterial disease patients	DULLAC Score	[32]
**Echocardiography**	Aortic valve Mitral annular and aortic root	Coronary disease patients	THC Score	[33]
**Transthoracic** **Echocardiography**	Coronary arteries	Coronary disease patients	Echo Score	[34]
**B-mode Doopler** **Ultrasound**	Carotid arteries, abdominal aorta and lower limbs vessels	Low-intermediate cardiovascular risk patients	CALC Score	[35]
**Intravascular Ultrasound**	Coronary arteries	Coronary disease patients	IVUS Calcium Score	[36]
**Molecular Imaging** **Techniques**				
**Electron Beam or** **Multislice Computed** **Tomography**	Coronary arteries	CKD patients	Agatston Score	[37]
	Coronary arteries	Asymptomatic patients	Agatston Score	[38,39,40]
	Coronary arteries	Coronary artery disease patients	Agatston Score	[41]
	Thoracic aorta	Peritoneal dialysis patients	Agatston Score	[38]
	Abdominal aorta	Asymptomatic patients	Agatston Score	[40]
	Mitral and aortic valve	CKD	Agatston Score	[42]
	Coronary arteries	Coronary calcification patients	Volume Score	[43]
	Coronary arteries	Asymptomatic patients	Mass Score	[44]

**Table 2:** Vascular Calcification Players Associated with Mineral Metabolism.

Promoters	Role in Vascular Calcification	Inhibitors	Role in Vascular Calcification
FGF-23 [80]	Hormone that functions as a central endocrine factor that regulates phosphate balance by modulating phosphate reabsorption in the kidney, parathyroid hormone (PTH) secretion and vitamin D metabolism.	Fetuin-A [84]	Binds directly to calcium and phosphate forming mineral complexes (Calciprotein Particles). It represses growth of crystal and mineral deposition.
OC [85]	Vitamin K-dependent protein (VKDP) secreted by osteoblasts functioning as a negative regulator of bone formation, regulator of mineral maturation rate and mechanical stabilizer of bone matrix; regulator of glucose metabolism.	MGP [86]	VKDP functioning as an inhibitor of soft tissue calcification involved in the VSMCs phenotypic differentiation, binding to calcium and calcium-phosphate circulating forms, thereby preventing its growth and matrix deposition.
Phosphate [87]	Promotes VSMC differentiation and apoptosis; elevates FGF-23; decreases Klotho expression; clusters with calcium in mineral complexes and promotes matrix mineralization.	GRP [46]	VKDP functioning as an inhibitor of soft tissue calcification involved in the VSMCs phenotypic differentiation and mineralization competence of extracellular vesicles; inhibitor of mineral crystal maturation and growth in blood; anti-inflammatory agent.
Calcium [88]	Clusters with phosphate in mineral complexes and promotes matrix mineralization; influences parathyroid hormone and Vitamin D production stimulus affecting FGF-23 levels.	OPN [89]	Prevents calcium crystal growth and accelerates osteoclast function.
		OPG [90]	Binds to the RANKL, thereby interfering with RANK-RANKL axis. Inhibition of RANKL-RANK inhibits the differentiation of osteoclast maturation, and thus, bone resorption.
		α-Klotho [79]	Klotho decreases kidney phosphate reabsorption by acting as a coreceptor for FGF23. Reduced levels are associated with cell senescence, cell apoptosis, oxidation induced cell damage and lower autophagy activity.
		Vitamin K [91]	Is the co-factor of γ-glutamyl carboxylase enzyme, responsible for the y-carboxylation of VKDPs.

FGF23, Fibroblast Growth Factor 23; OC, Osteocalcin; MGP, Matrix Gla Protein; GRP, Gla Rich Protein; OPN, Osteopontin; OPG, Osteoprotegerin; VKDPs, Vitamin K-Dependent Proteins; VSMCs, Vascular Smooth Muscle Cells; RANKL, Receptor Activator of Nuclear Factor Kappa Beta.

**Table 3:** Vascular Calcification Players and their Correlation with Calcification Scores from CKD Clinical Studies.

Clinical Markers	Population	VC Detection Method	Evidence of VC Marker and Score Association	Refs.
Phosphate	CKD	CAC	Yes	[97]
	Nondialysis CKD	Kauppila and Adragão	Yes	[98]
	Nondialysis CKD	Kauppila and Adragão	No	[99]
GF23	CKD	Adragão	Yes	[46]
	Hemodialysis	CAC-Agatston	Yes	[100]
	Hemodialysis	Echocardiography	No	[89]
	Hemodialysis	CAC-Agatston	Yes	[101]
α-Klotho	Hemodialysis	CAC	Yes	[102]
OPN	Hemodialysis	Echocardiography	Yes	[89]
OC	Hemodialysis	Transthoracic Echocardiography	No	[103]
OPG	Hemodialysis	CAC-Agatston	Yes	[90]
	Hemodialysis	CAC-Agatston	Yes	[100]
Vitamin K status/ dp-ucMGP	Hemodialysis	CAC-Agatston	No	[104]
	CKD	CAC-Agatston	No	[105]
	Hemodialysis	CAC-Agatston	No	[106]
	CKD	CAC	Yes	[86,107]
	Hemodialysis	Kauppila	Yes	[108]
	Hemodialysis	CAC-Agatston	No	[100]
GRP	CKD	Adragão	Yes	[46]
	CVD	CAC	No	[109]
	CKD	Ultrasound	Yes	[110]
	CKD	Echocardiography	Yes	[111]
Fetuin-A	Renal Transplant	CAC	Yes	[112]
	ESRD	CAC	No	[113]
	CKD	CAC	Yes	[84]

CAC, Coronary Artery Calcium; CKD, Chronic Kidney Disease; FGF-23, Fibroblast Growth Factor 23; OC, Osteocalcin; MGP, Matrix Gla Protein; GRP, Gla Rich Protein; OPN, Osteopontin; OPG, Osteoprotegerin.

## Data Availability

Not applicable.
